# Imatinib Enhances Functional Outcome after Spinal Cord Injury

**DOI:** 10.1371/journal.pone.0038760

**Published:** 2012-06-19

**Authors:** Mathew B. Abrams, Ingrid Nilsson, Sebastian A. Lewandowski, Jacob Kjell, Simone Codeluppi, Lars Olson, Ulf Eriksson

**Affiliations:** 1 Department of Neuroscience, Karolinska Institutet, Stockholm, Sweden; 2 Department of Medical Biochemistry and Biophysics, Karolinska Institutet, Stockholm, Sweden; The University of Western Australia, Australia

## Abstract

We investigated whether imatinib (Gleevec®, Novartis), a tyrosine kinase inhibitor, could improve functional outcome in experimental spinal cord injury. Rats subjected to contusion spinal cord injury were treated orally with imatinib for 5 days beginning 30 minutes after injury. We found that imatinib significantly enhanced blood-spinal cord-barrier integrity, hindlimb locomotor function, sensorimotor integration, and bladder function, as well as attenuated astrogliosis and deposition of chondroitin sulfate proteoglycans, and increased tissue preservation. These improvements were associated with enhanced vascular integrity and reduced inflammation. Our results show that imatinib improves recovery in spinal cord injury by preserving axons and other spinal cord tissue components. The rapid time course of these beneficial effects suggests that the effects of imatinib are neuroprotective rather than neurorestorative. The positive effects on experimental spinal cord injury, obtained by oral delivery of a clinically used drug, makes imatinib an interesting candidate drug for clinical trials in spinal cord injury.

## Introduction

Traumatic injury to the spinal cord results in irreversible locomotor, sensory, and autonomic dysfunctions for which there is currently no effective pharmacologic treatment. The functional losses are due not only to the initial physical disruption of spinal cord tissue and vascular changes, but also to a cascade of secondary events triggered by the initial insult, which results in further damage to the spinal cord at and around the site of injury [1]. These detrimental secondary events include the loss of microcirculation, edema, hemorrhage, and local inflammation [2], [3], [3], [4]. Incomplete understanding of how these events evolve and contribute to pathology has been a major impediment to the development of treatments for spinal cord injury.

Imatinib (Gleevec®, Novartis) is a tyrosine kinase inhibitor used clinically to treat Bcr/Abl-expressing leukemias and c-Kit-expressing gastrointestinal stromal tumors. Experimentally, imatinib has been reported to modulate tyrosine kinase signaling cascades involved in local inflammation, such as c-Kit, c-Fms, and LCK [5], [6]. Imatinib-induced inhibition of c-Kit and c-Fms activation has been reported to inhibit cytokine production in mast cells and macrophages, respectively [5], while inhibition of LCK has been reported to modulate the cytokine production/secretion profile in effector T cells [7]. Imatinib has also been reported to inhibit PDGF signaling [8], [9], [10] and, in an experimental model of ischemic stroke, to reduce edema and hemorrhage [8], [11]. In the stroke model, evidence was obtained for a mechanism involving normalization of the blood-brain barrier by imatinib-induced inhibition of PDGFR-α signaling on perivascular astrocytes [8].

Since imatinib has previously been shown to reduce hemorrhage, edema, and inflammation, which are all considered detrimental to functional outcome in spinal cord injury [12], [13], [14], [15], [16], [17], [18], [19], we investigated the effects of imatinib on functional and histological outcome in a clinically relevant experimental model of spinal cord injury in rats. We found that an oral, 5-day treatment course with imatinib significantly enhanced hind limb locomotor function, bladder function, and sensorimotor integration. We also found that imatinib significantly enhanced tissue preservation and reduced inflammation, astrogliosis, and chondroitin sulfate proteoglycan deposition. Furthermore, we found evidence in support of the potential of imatinib to normalize vascular integrity in the injured spinal cord. Our results provide evidence in support of the therapeutic potential of imatinib as a possible treatment for spinal cord injury.

## Results

### Imatinib Enhances Functional Outcome

**Figure 1:**
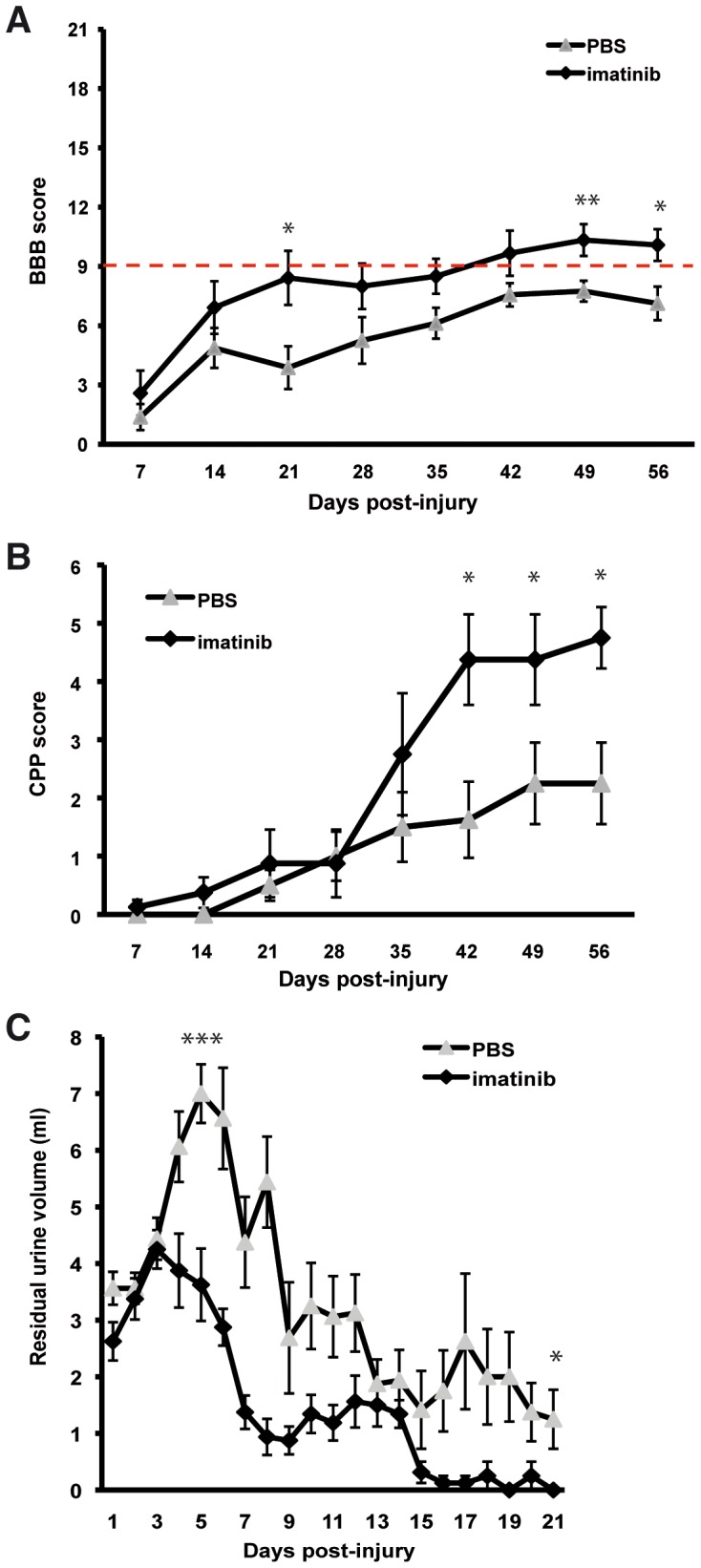
Imatinib enhances functional outcome. (**A**) Hindlimb locomotor function assessed by the BBB locomotor rating scale. The red line indicates BBB score 9, when weight-support begins. Scores above 9 represent walking, while scores below 9 represent no walking. (**B**) Contact plantar placement (CPP) test of sensorimotor function. (**C**) Bladder residual urine volumes. Imatinib (N = 10) compared to PBS (N = 10). Data presented as mean ± SEM: *P<0.05, **P<0.01, and ***P<0.001.

We investigated the effects of a 5-day treatment course with either imatinib (250 mg/kg/day orally) or PBS beginning 30 minutes after injury on functional outcome measures in rats subjected to a contusion injury to the spinal cord (25 mm weight drop). Assessment of hindlimb function revealed significantly enhanced locomotor function. By day 56 post-injury, 70% of imatinib-treated rats had regained the ability to make uncoordinated weight-supported steps (mean ± SD: 10.3±1.6; N = 10; Fig. 1 A, Fig. S1), while PBS-treated rats only regained the ability to extensively move all three hindlimb joints (mean ± SD: 7.1±1.43; N = 10; Fig. 1 A, Fig. S1). Using the contact plantar placement test, we found that sensorimotor integration was significantly better with imatinib (mean ± SD: 4.7±1.33; N = 10) than PBS (mean ± SD: 2.2±1.9; N = 10) by day 56 post-injury (Fig. 1 B, Fig. S1). Assessment of bladder function revealed that imatinib significantly reduced peak residual urine volumes (mean ± SD: 4.25±0.85 ml; N = 10) compared to PBS (mean ± SD: 7.0±1.29 ml; N = 10) and expedited restoration of voiding function (Fig. 1 C, Fig. S1). By day 21 post-injury, 0.15±0.33 ml of urine (mean ± SD) was collected from imatinib-treated rats compared to 1.2±0.85 ml of urine (mean ± SD) from PBS-treated rats. This effect was not due to altered fluid intake, since fluid consumption did not differ between imatinib and PBS treated groups (Data not shown). In addition, imatinib treatment was associated with less injury-induced weight-loss than PBS treatment (imatinib, N = 10; PBS, N = 10; Fig. S2).

### Imatinib Enhances Tissue Preservation

**Figure 2:**
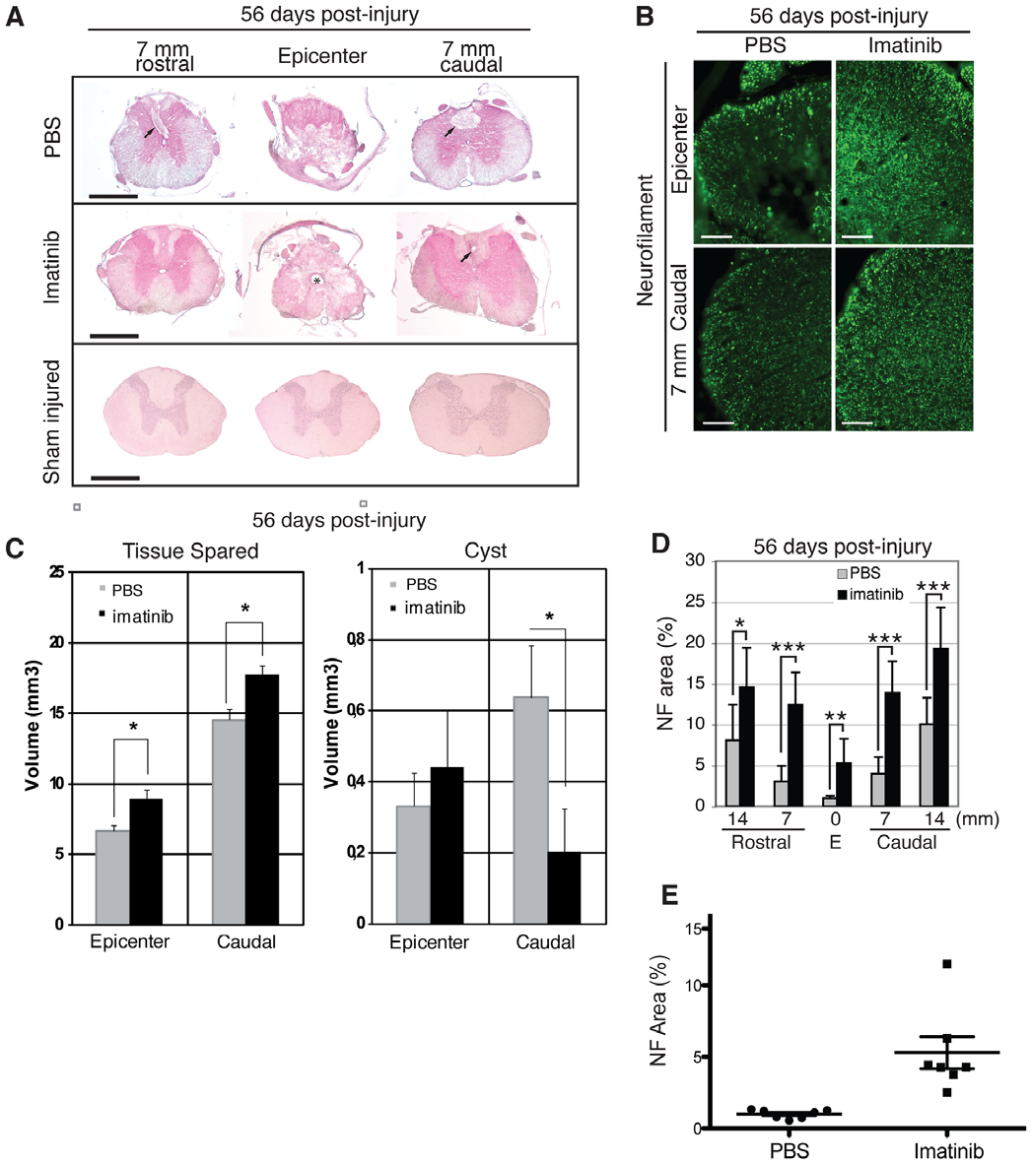
Imatinib enhances tissue preservation. H&E staining was used to visualize tissue and spinal cysts within the epicenter and in spinal segments 7 and 14 mm rostral and caudal to the injury. (**A**) Representative micrographs of tissue preservation and spinal cysts at day 56 post-injury in the injury site and in spinal segments 7 mm rostral and caudal to the injury. Arrows indicate spinal cysts. Asterisk indicates possible enlargement of the central canal. (**B**) Representative micrographs of NF immunoreactivity within the injury site at day 56 post-injury. (**C**) Quantification of tissue preservation and spinal cyst volumes within the injury site and 7 mm caudal to the epicenter at day 56 post-injury (imatinib n = 10; PBS n = 10). (**D**) Quantification of NF immunoreactivity within the injury site and in spinal segments 7 and 14 mm rostral and caudal to the injury at day 56 post-injury. (**E**) Scatter plot of NF immunoreativity within the injury site. Imatinib (N = 7) compared to PBS (N = 7). Data presented as mean ± SD: *P<0.05, **P<0.01, and ***P<0.001. Scale bars: (**A**) 1 mm, (**B**) 100 µm.

Imatinib led to significantly more preserved tissue at the injury site, as well as in spinal segments caudal to the injury, as compared to PBS-treated rats when assessed at day 56 post-injury (imatinib, N = 7; PBS, N = 7; Fig. 2 A, C); and imatinib was associated with increased myelin volume (2.010±0.49%, N = 7) compared to PBS (1.413±0.43%, N = 7; p = 0.0504, Fig. S3). Imatinib also significantly reduced spinal cyst volumes caudal to the injury (imatinib, N = 7; PBS, N = 7; Fig. 2 A, C). Importantly, imatinib was associated with a significant increase in the area of neurofilament immunoreactive axons within the white matter of the injury site (mean ± SD: 5.3±2.95%) compared to PBS treatment (mean ± SD: 1.0±0.29%), as well as in spinal segments 7 and 14 mm rostral and caudal to the injury (imatinib, N = 7; PBS, N = 7; Fig. 2 B, D, E).

### Imatinib Treatment Modulates the Inflammatory Response

**Figure 3:**
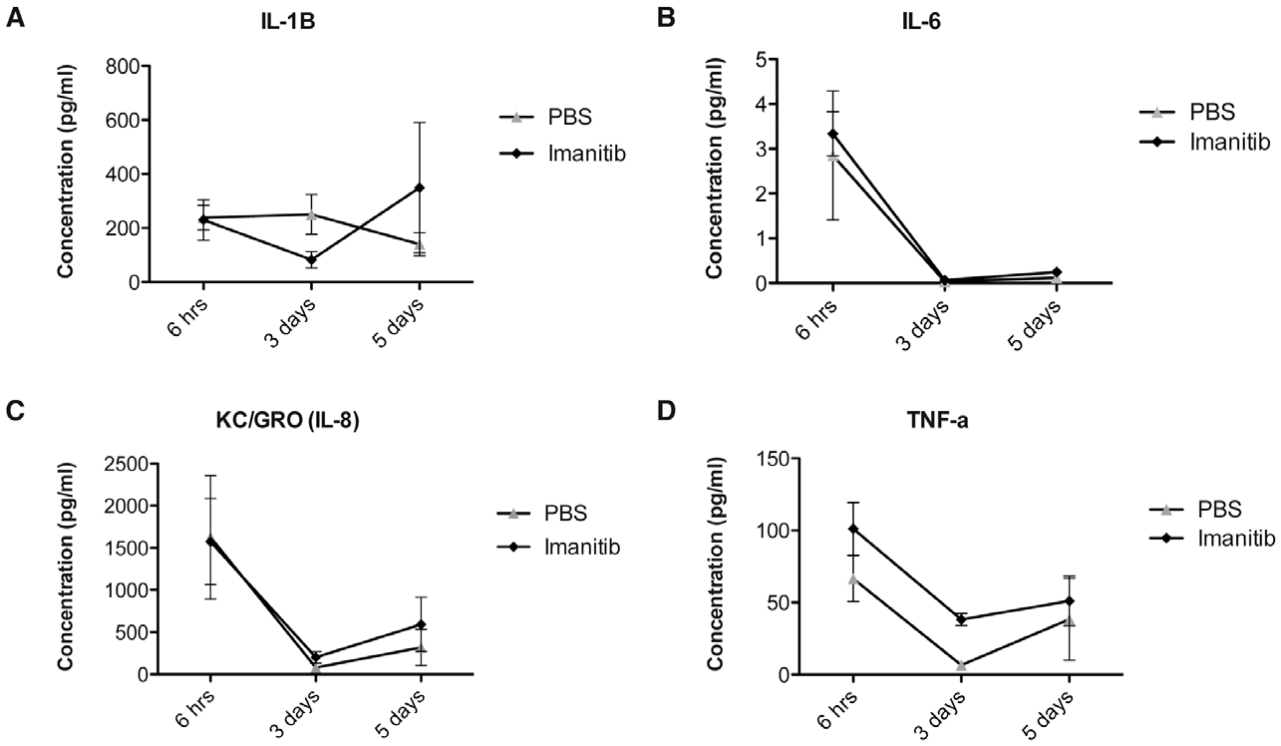
Impact of imatinib on plasma concentrations of pro-inflammatory cytokines. (**A**) IL-1β plasma concentrations. (**B**) IL-6 plasma concentrations. (**C**) IL-8 plasma concentrations. (**D**). TNF-α plasma concentrations. Imatinib (N = 4) compared to PBS (N = 4). Data presented as the mean ± SD. ***P<0.001.

**Figure 4:**
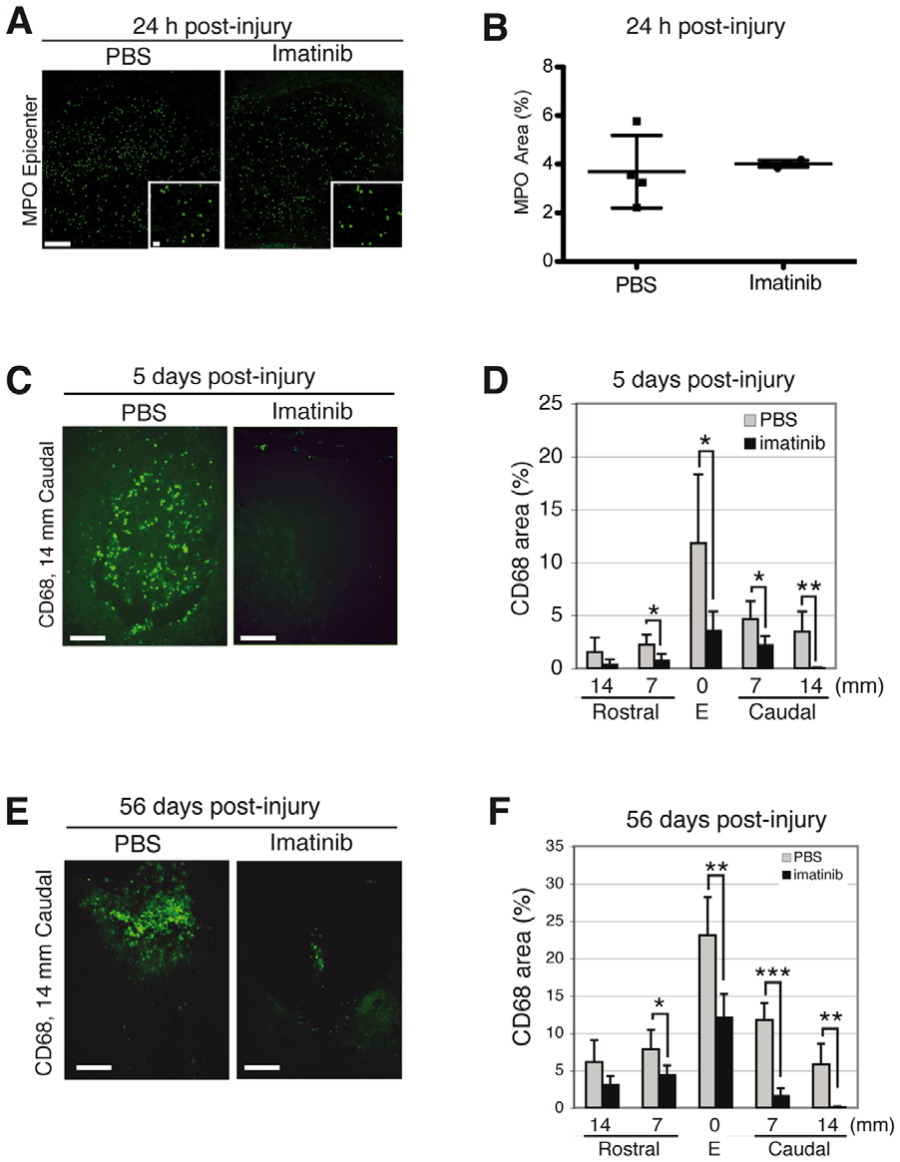
Imatinib attenuates acute and chronic inflammation. Effects of 30 minute delayed imatinib treatment on MPO immunoreactive neutrophils within the injury site at 24 hours post-injury and CD68 immunoreactive activated macrophages/microglia at day 5 post-injury. (**A**) Representative micrographs of MPO immunoreactivity at 24 hours post-injury. (**B**) Quantification of MPO immunoreactivity within the injury site at 24 hours post-injury (imatinib, N = 4; PBS, N = 4). (**C**) Representative micrographs of CD68 immunoreactivity at day 5 post-injury. (**D**) Quantification of CD68 immunoreactivity within the spinal cord at day 5 post-injury (imatinib, N = 4; PBS, N = 4). (**E**) Representative micrographs of CD68 immunoreactivity for activated macrophages/microglia within the injury site 14 mm caudal of the epicenter at day 56 post-injury. (**F**) Quantification of the relative area occupied by CD68 immunoreactivity within the injury site and in spinal segments 7 and 14 mm rostral and caudal to the injury at day 56 post-injury (imatinib, N = 6; PBS, N = 6). Data presented as mean ± SD: *P<0.05, **P<0.01, and ***P<0.001. Scale bars: 100 µm, inset: 10****µm.

We assessed plasma concentration of IL-1β, IL-6, IL-8, and TNF-α, cytokines known to elicit the acute phase response, and found that imatinib had no significant impact on any of these cytokines at 6 hours and 5 days post-injury (imatinib, N = 4; PBS, N = 4; Fig. 3). Imatinib did lead to a significant increase in plasma levels of TNF-α compared to PBS (38.18±7.2 pg/ml vs. 6.53±3.9 pg/ml) at 3 days post-injury (p<0.001; Fig. 3). We next assessed the effects of imatinib on neutrophil infiltration into the injury site 24 hours after injury using MPO immnoreactivity and found that imatinib did not significantly alter MPO immunoreactivity (imatinib, N = 4; PBS, N = 4; Fig. 4 A, B).

CD68 immunoreactivity was used to detect activated microglia/macrophages at day 5 post-injury to assess the impact of the 5-day treatment course on inflammation. We found that imatinib treatment significantly reduced the areas of CD68 immunoreactive activated macrophages/microglia within the injury site (mean ± SD: 3.56±1.88%) as compared to PBS treatment (mean ± SD: 12.05±6.34%), as well as in spinal segments around the injury (imatinib, N = 4; PBS, N = 4; Fig. 3 C, D). Imatinib treatment was also associated with significantly reduced CD68 immunoreactivity at day 56 post-injury within the injury site (mean ± SD: 12.07±3.15) compared to PBS treatment (mean ± SD: 23.09±5.09%; imatinib, N = 7; PBS, N = 7; Fig. 3 E, F). We next co-labeled CD68 positive cells with the proliferation marker Ki67, and found that imatinib had no significant impact on proliferation of CD68 positive cells as studied at day 5 post-injury (imatinib, N = 4; PBS, N = 4; Table 1; Fig. S4). We also investigated whether imatinib affected the proliferation of leukocytes expressing CD11b, and found that there was no significant effect (imatinib, N = 4; PBS, N = 4; Table 1; Fig. S4). Co-localization of CD68 at day 7 post-injury with either PDGFR-α or NG2 revealed that CD68 immunoreactive cells did not co-localize with either marker (Fig. S5). We did, however, find a small population of CD11b immunoreactive cells that were co-localized with NG2 (Fig. S4).

**Table 1:** Proportion (%) of proliferating cells co-expressing CD68, CD11b, PDGFRs or NG2 at the injury site and 7 mm caudal to the injury.

	Injury site		7 mm caudal to injury	
	PBS	imatinib	PBS	imatinib
CD68	8.7±3.2	8.8±5.4	13.3±7.9	10.4±10.0
CD11b	36.4±5.2	31.2±7.8	42.9±8.6	48±5.3
PDGFR-α	13.4±9.6	20.8±8.9	10.8±7.8	23.3±11.8
PDGFR-β	8.1±2.7	1.6±3.1*	2.1±2.4	0
NG2	50.4±9.4	45.0±4.1	50.7±11.7	17.9±13.7*

Data presented as mean ± SD: *P<0.05.

### Imatinib Attenuates Astrocyte Reactivity and Chondroitin Sulfate Proteoglycan Deposition

**Figure 5:**
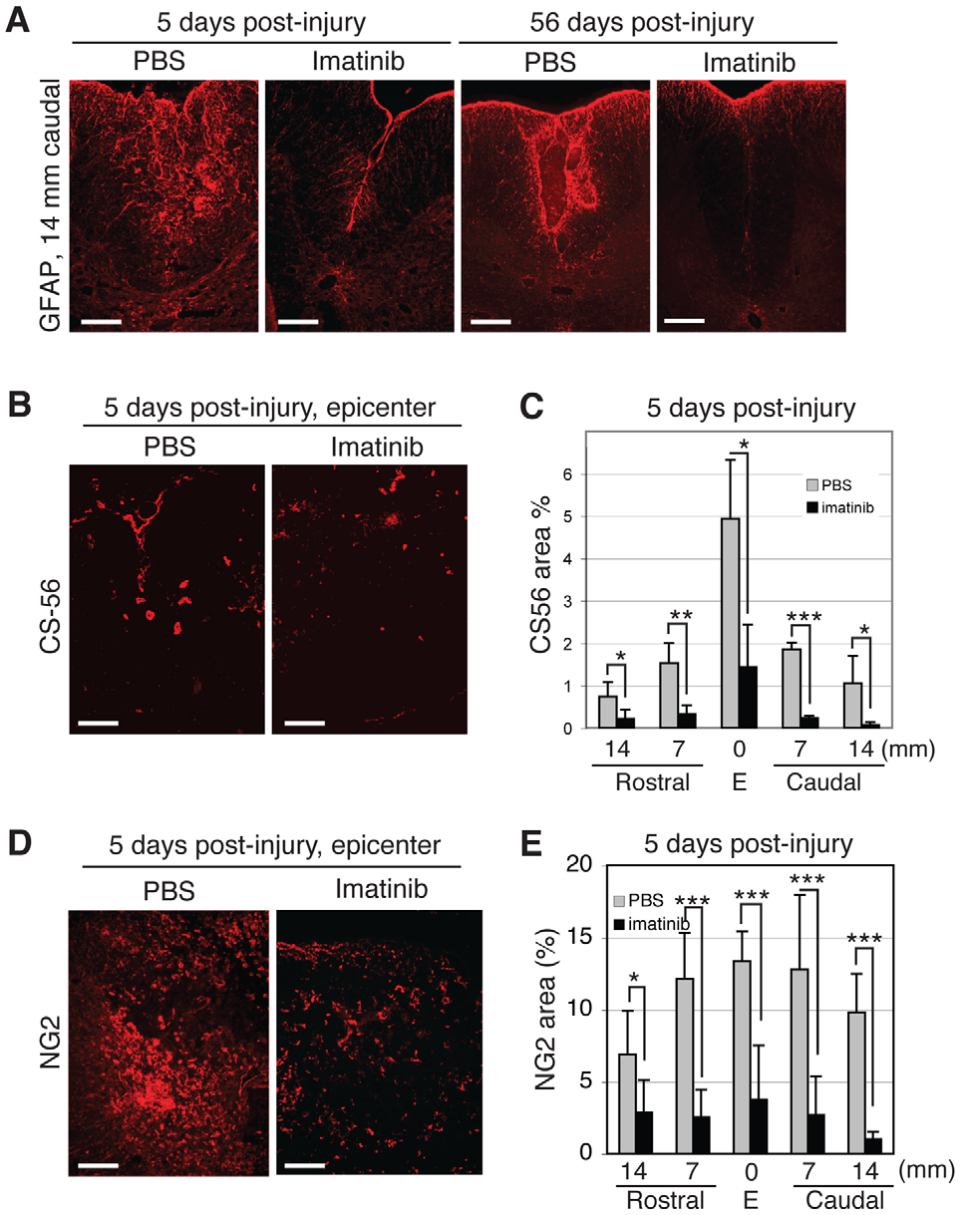
Imatinib attenuates astrogliosis and CSPG deposition. (**A**) Representative micrographs of dorsomedial GFAP immunoreactivity 14 mm caudal to the injury site at 5 and 56 days post-injury. (**B**) Representative micrographs of CS56 immunoreactivity within the injury site at day 5 post-injury. (**C**) Quantification of CS56 immunoreactivity within the injury site and spinal segments 7 and 14 mm rostral and caudal to the injury at day 5. (**D**) Representative micrographs of NG2 immunoreactivity within the injury site at day 5 post-injury. (**E**) Quantification of NG2 immunoreactivity within the injury site and in spinal segments 7 to 14 mm rostral and caudal to the injury site at day 5. Imatinib (day 5, N = 4; day 56, N = 6) compared to PBS (day 5, N = 4; day 56, N = 6). Data presented as mean ± SD: *P<0.05, **P<0.01, and ***P<0.001. Scale bars: 100 µm.

Imatinib was associated with less intense GFAP immunoreactivity than PBS at day 5 (imatinib, N = 4; PBS, N = 4; Fig. 5 A). While GFAP intensity increased between days 5 and 56, it remained less intense in imatinib-treated, compared to PBS-treated rats (imatinib, N = 7; PBS, N = 7; Fig. 5 A) also 56 days after injury. To study chondroitin sulphate proteoglycans, we used CS56 and NG2 antibodies and found that imatinib significantly reduced the area of CS56 and NG2 immunoreactivity within the injury site and in spinal segments 7 and 14 mm rostral and caudal to the injury at day 5 post-injury (imatinib, N = 4; PBS, N = 4; Fig. 5 B-E, Fig. S6). CS56 and NG2 immunoreactivities within the injury site were 1.83±1.11 and 3.74±3.77, respectively, in the spinal cords of imatinib-treated rats and 4.93±1.12 and 13.33±2.09 in the spinal cords of PBS-treated rats. As NG2 is also a marker for progenitor cells capable of differentiating into oligodendrocytes and astrocytes, we quantified the effect of imatinib on the proliferation of NG2 immunoreactive cells by double labeling with Ki-67. We found that imatinib significantly reduced the percentage of NG2/Ki-67double-labeled cells 7 mm caudal to the injury, with 17.9±13.7% proliferating NG2 positive cells in the spinal cords of imatinib-treated rats compared to 50.7±11.7% proliferating NG2 positive cells in the spinal cords of PBS-treated rats (imatinib, N = 4; PBS, N = 4; Table 1; Fig. S4).

### Imatinib Enhances Blood-spinal Cord-barrier Integrity

**Figure 6:**
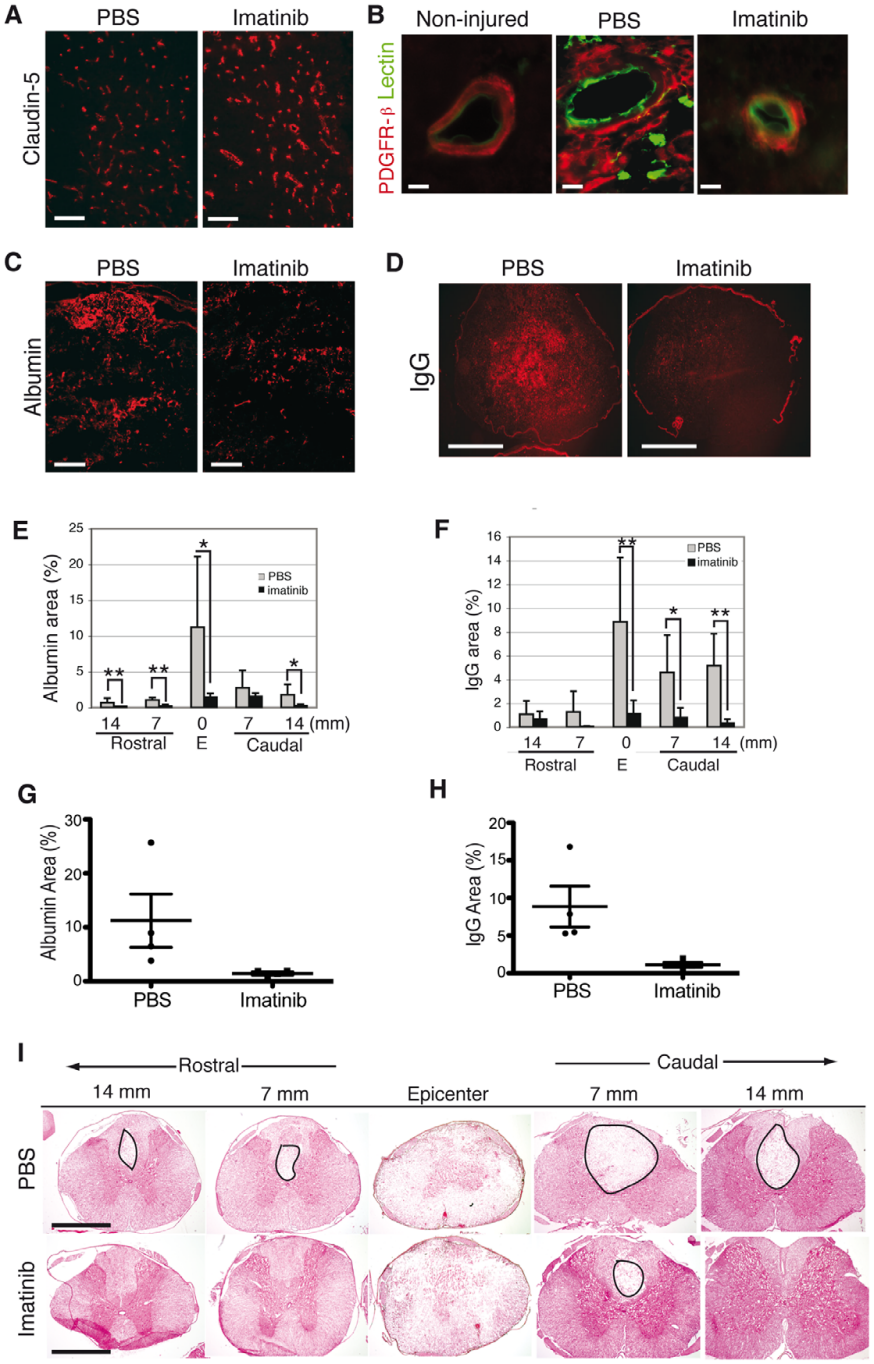
Imatinib enhances vascular integrity. (**A**) Tight junction protein, claudin-5, immunoreactivity in endothelial cells in the dorsal horn at 5 days post-injury is stronger in imatinib than in PBS-treated rats. This indicates better preservation of the BSCB integrity. (**B**) Representative micrographs of PDGFR-β/lectin immunoreactivity at day 5 post-injury in the spinal cords of non-injured, PBS-treated, and imatinib-treated rats. (**C**) Representative micrographs of albumin immunoreactivity within the injury site at day 5 post-injury. (**D**) Representative micrographs of IgG immunoreactivity within the injury site at day 5 post-injury. (**E**) Quantification of albumin immunoreactivity within the spinal cord at day 5 post-injury. (**F**) Quantification of IgG immunoreactivity within the spinal cord at day 5 post-injury. (**G**) Scatter plot of the area of albumin immunoreactivity within the injury site at day 5 post-injury. (**H**) Scatter plot of the area of IgG immunoreactivity within the injury site at day 5 post-injury. (**I**) H and E staining of the spinal cord at day 5 post-injury. Circles indicate areas without H and E staining. Imatinib (day 5, N = 4) compared to PBS (day 5, N = 4). Data presented as mean ± SD: *P<0.05, **P<0.01, and ***P<0.001. Scale bars: (**A,C,E**) 100 µm,(**G,I**) 1 mm, (**B**) 10 µm.

To determine the effects of imatinib on blood-spinal cord-barrier (BSCB) integrity, we evaluated the expression of claudin-5, an endothelial cell tight junction protein at day 5 after injury. We found more intense claudin-5 immunoreactivity within the dorsal horns of imatinib-treated rats compared to PBS-treated rats (imatinib, N = 4; PBS, N = 4; Fig. 6 A). We also observed that imatinib caused pericytes, as identified by PDGFR-β immunoreactivity, to remain confined to the vascular walls after injury, thus resembling the distribution of PDGFR-β expressing cells in non-injured tissue. In contrast, spinal cord injury caused PDGFR-β immunoreactive cells to expand their territory away from the blood vessels in PBS-treated rats (imatinib, N = 4; PBS, N = 4; Fig. 6 B).

To assess BSCB integrity, we quantified leakage of two proteins, albumin and IgG, which are normally contained within the circulation but leak into parenchyma following CNS injury. At day 5 post-injury, we found that imatinib significantly reduced leakage of both proteins into the injury site (1.44±0.56 and 1.12±0.65, respectively) compared to PBS treatment (11.23±9.8% and 8.86±5.43%, respectively). Imatinib also significantly reduced albumin leakage into spinal cord segments rostral and caudal to the injury and reduced IgG leakage in spinal segments caudal to the injury (imatinib, N = 4; PBS, N = 4; Fig. 6 C-H). Furthermore, early signs of better tissue morphology were observed in the spinal cord of imatinib-treated rats at day 5 post-injury (Fig. 6 I).

### Imatinib Reduces Expansion and Proliferation of PDGFR Expressing Cells

**Figure 7:**
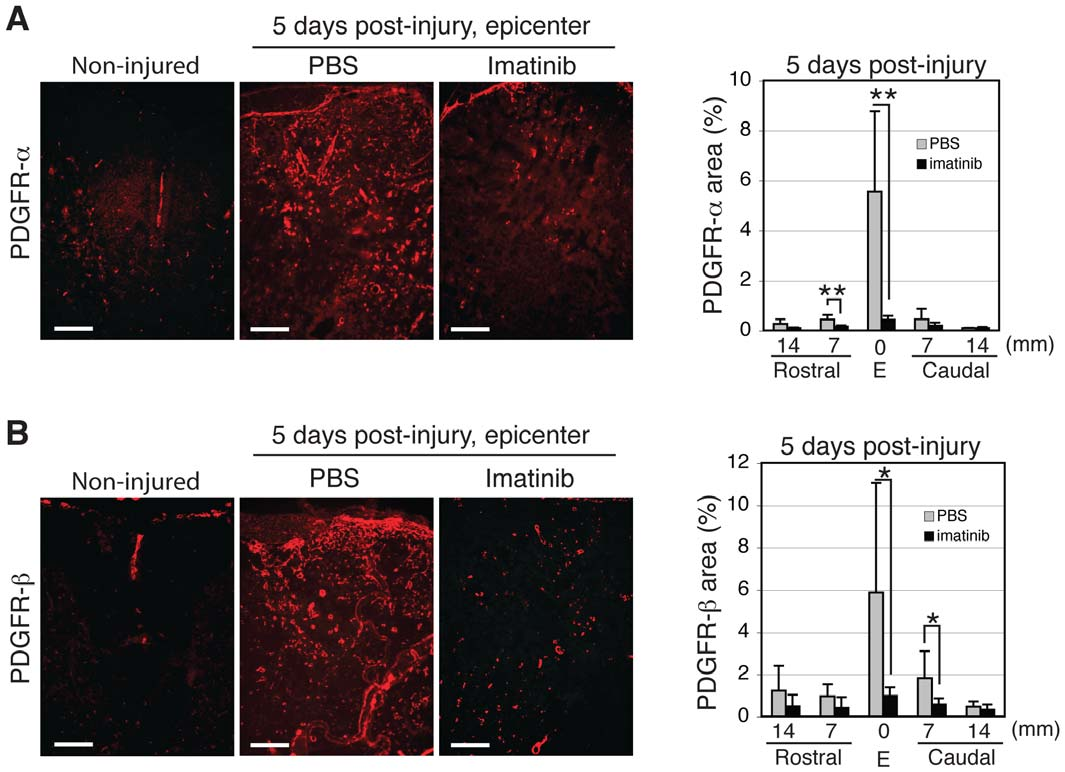
Effects of 30 minute delayed, 5-day imatinib treatment on PDGFR expression at day 5 after contusion spinal cord injury. (A) Representative micrographs of PDGFR-α immunoreactivity within the injury site and quantification of PDGFR-α immunoreactivity within the injury site and in spinal segments rostral and caudal to the injury in non-injured, PBS-treated, and imatinib-treated rats. (B) Representative micrographs of PDGFR-β expression within the injury site and quantification of PDGFR-β expression within the injury site and spinal segments rostral and caudal to the injury in non-injured, PBS-treated, and imatinib-treated rats. Imatinib (N = 4) compared to PBS (N = 4). Data presented as mean ± SD: *P<0.05. Scale bars: 100 µm.

In the uninjured spinal cord, PDGFR-α is expressed on perivascular astrocytic endfeet (N = 3; Fig. S7), while PDGFR-β is expressed on pericytes. Spinal cord injury induced a rapid increase of both PDGFR-α and PDGFR-β expression, evident 24 hours after injury (sham, N = 2; injured, N = 2; Fig. S7). At day 5 post-injury, we observed a significant reduction in PDGFR-α immunoreactivity within the dorsal column at the injury site and 7 mm rostral to the injury in the spinal cords of imatinib-treated (2.53±0.06) rats when compared to the spinal cords of PBS-treated rats (4.59±1.14; imatinib, N = 4; PBS, N = 4; Fig. 7 A). Similarly, we observed a significant reduction in PDGFR-β immunoreactivity within the dorsal column of imatinib-treated vs PBS-treated rats, 1.95±0.64% and 3.18±1.33% respectively (imatinib, N = 4; PBS, N = 4; Fig. 7 B). We also investigated whether PDGFRs were expressed constitutively on neurons as previously reported [20] and found that PDGFRs were neither constitutively expressed nor induced by injury in neurons in the rat spinal cord (sham, N = 2; injured, N = 2; Fig. S8).

Co-labeling with the proliferation marker, Ki67, revealed that only a small population of PDGFR-α and PDGFR-β immunoreactive cells were proliferating within the injury site and 7 mm caudal to the injury at day 5 post-injury (imatinib, N = 4; PBS, N = 4; Table 1, Fig. S4). Nevertheless, imatinib led to a significant reduction of the small number of proliferating PDGFR-β immunoreactive cells within the injury site (1.6±3.1 vs 8.1±2.7 for imatinib and PBS respectively). Imatinib had no effect on the proliferation of PDGFR-α immunoreactive cells within the injury site or 7 mm caudal to the injury at day 5 post-injury.

## Discussion

We found that an oral, 5-day treatment with imatinib significantly enhanced functional outcome after experimental contusion spinal cord injury of the spinal cord. Imatinib-treated rats regained the ability to make weight-supported steps with their hind limbs, which was in stark contrast to PBS-treated rats that only regained the ability to make rhythmic sweeping motions with their hind limbs. Imatinib-treated rats also regained significantly greater sensorimotor integration than PBS-treated rats. Injury-induced bladder dysfunction was restored by imatinib, and injury-induced weight loss was reduced in imatinib-treated rats. The restoration of bladder function obtained with imatinib is of particular clinical relevance for quality of life, since restoration of bladder function is consistently ranked as a higher priority than walking for both quadriplegics and paraplegics [21].

Currently, high dose methylprednisolone administered within 8 hours of injury is the only clinically used pharmacologic intervention for spinal cord injury. Controversy regarding its efficacy has led clinics to abandon its use [22], so there is need for alternative pharmacologic interventions for the treatment of spinal cord injury. The beneficial effects of imatinib on functional outcome presented here indicate that imatinib may potentially fill this need.

The beneficial effects of imatinib on functional outcome may in part be due to increased tissue preservation within the injury site, since the amount of preserved tissue has previously been positively correlated with functional outcome [23]. In particular, the significantly larger amount of NF-reactive axons traversing the injury site may have also contributed to the functional effects, since the number of axons (protected and/or regenerated) traversing the epicenter is arguably the single most important histological indication of degree of function. Imatinib also counteracted other indices of tissue pathology, such as astrogliosis and CSPG deposition, which is presumably valuable because both the astroglial scar and CSPGs are considered detrimental to axon regeneration and functional outcome [24], [25], [26].

The expansion of NG2 immunoreactive progenitor cells, which differentiate into both astrocytes and oligodendrocytes in response to injury [27], was significantly reduced by imatinib. This observation is significant in that NG2 cells have been reported to proliferate in response to demyelination [28], [29], and the time course of NG2 cell proliferation has been reported to parallel the time of demyelination and remyelination in the spinal cord [30]. It is also important to mention that little to no activated macrophages/microglia and very few non-activated macrophages/microglia expressed this marker, so the effects of imatinib on NG2 immunoreactive cells does not reflect an effect on the subpopulations of macrophages/microglia that have been reported to express this marker [31], [32]. Thus, it is plausible that the imatinib-induced reduction of NG2 cell proliferation reflects decreased tissue damage and the observed reduction in the amount of reactive astrocytes.

Imatinib has been reported to inhibit multiple receptor tyrosine kinases in mast cells, macrophages, and T-lymphocytes, as well as PDGFR-α and β [5], [33], [34]. We observed a significant reduction in amounts of activated macrophages/microglia in the spinal cords of imatinib-treated rats, which is noteworthy because depletion of hematogenous macrophages and attenuation of T-lymphocytes, potent activators of macrophages, have both been reported to enhance tissue preservation in experimental spinal cord injury [35], [36]. Moreover, blockade of α4β1 integrin has been reported to limit secondary inflammatory damage after spinal cord injury [37], [38], [39] Imatinib has also been shown to abrogate macrophage activation and cytokine production in an experimental arthritis model [5]. Under the current experimental conditions, imatinib had no significant effect on ED-1 immunoreactive macrophage proliferation; however, we cannot rule out that imatinib had an effect on activation state or cytokine production.

We observed that imatinib markedly reduced the expansion of PDGFR-α and PDGFR-β expressing cell populations, with the strongest reductions being seen in the areas of the greatest tissue preservation. Astrocytes and O2A progenitors, as well as pericytes expressing PDGFR-α and PDGFR-β, respectively, are sources of CSPGs [40], [41]. Thus, we have observed not only a marked reduction in CSPG, but also a marked reduction of cellular sources of CSGP. Although it has been reported that PDGFR-α is expressed on neurons in primary neuronal cultures [20], PDGFR-α was neither constitutively expressed nor induced by injury in neurons in the rodent spinal cord, indicating that PDGFR-α inhibition is not having a direct effect on neurons. It has also been reported that a subset of activated macrophages/microglia express PDGFR-α [42]; however, under the current experimental condition, ED-1 immunoreactive activated macrophages/microglia did not express PDGFR-α. Thus, the effects of imatinib on PDGFR-α do not constitute an effect of imatinib on the subpopulation of activated macrophages/microglia that express PDGFR-α.

We found that imatinib increased immunolabeling of the endothelial tight junction protein, claudin-5, and that PDGFR-β immunoreactive pericytes remained located tightly around vessel walls in a pattern similar to that seen in non-injured rats, thus indicating normalization of the blood-spinal cord-barrier. In the absence of imatinib, injury caused a spreading of PDGFR-β immunoreactive cells away from vessel walls. This observation is in accordance with a previous report that pericytes migrate away from the neurovascular unit in response to CNS injury [43]. The effects of imatinib on BSCB integrity are in agreement with the effects found in a mouse model of ischemic stroke, where imatinib promotes BBB integrity and reduces stroke volume by inhibiting PDGF-CC induced activation of PDGFR-α on perivascular astrocytic endfeet [8].

Given that imatinib has been reported to modulate immune cell function and secretion profile, it was surprising to observe that imatinib treatment had no significant impact on plasma concentrations of pro-inflammatory cytokines, IL-β, IL-6, IL-8, and TNF-α, reported to be involved in the orchestration of the inflammatory response [44], [39], [37] at 6 hours post-injury which translates to 5.5 hours after the treatment began. It was also surprising to find that imatinib had no impact on neutrophil infiltration into the injured spinal cord 24 hours after injury. Collectively, these results indicate that imatinib has no significant impact on the generation of the innate immune response and that the immune response between the treatment groups immediately after injury were similar.

Remarkably, imatinib treatment associated with increased plasma concentrations of TNF-α, an inflammatory cytokine produced primarily by macrophages and reported to induce inflammation [45], at day 3 post-injury. This was followed by significantly reduced areas of activated macrophage/microglia expression at day 5 post-injury, an unexpected finding since macrophages filtration has been reported to peak between 5 to 7 days [46]. One possible explanation for the lack of accordance between the plasma correlate of inflammation and tissue inflammation could be that of normalization of BSCB integrity in the spinal cords of rats treated with imatinib prevented macrophage infiltration into the injured spinal cord. Indeed, it has previously been reported that imatinib normalizes the integrity of the BBB and reduces infarct volume in a murine model of ischemia stroke [8]. Continued research is necessary to determine whether imatinib is acting directly on the BSCB or on immune cells, since inhibition/modulation of the secretion profile of immune cells could theoretically result in reduced permeability of the BSCB and normalization of blood vessels, while normalization of the BSCB by inhibiting immune cells from entering the spinal cord.

In conclusion, we present robust evidence in support of a new indication for imatinib as an acute treatment for spinal cord injury. The rapid time course of the beneficial effects and the fact that a 5-day treatment period leads to lasting improvements of both structure and function, suggest that imatinib is neuroprotective rather than neurorestorative. A specific advantage of imatinib is that it is currently in clinical use for other indications, offering a putative off the shelf therapy with fewer hurdles en route to clinical trials.

## Materials and Methods

### Ethics Statement

Animal work was approved by the North Stockholm Animal Ethics Committee (permit # N429/09). Animals received analgesic pre-operative, for 3 days post-injury to alleviate pain, and as needed thereafter. Antibiotics were given for 7 days post-injury to prevent bladder infection. Bladders were manually expressed twice/day until bladder function was restored. Animals were communally housed and had access to food/water at will.

### Spinal Cord Injury

Under deep anesthesia (Isofluorane), a laminectomy was performed at T10 and the caudal half of T9, and a 10 g weight was dropped from a height of 25 mm (*220 g,* Scanbur, Sweden) onto the dorsal surface of the spinal cord using the Impactor (Keck Center for Neurosciences) in female rats. The rats were randomly divided into 2 treatment groups, imatinib (N = 18) or PBS (N = 18). 250 mg/kg/day of imatinib was administered orally 30 minutes after injury and twice daily thereafter for 5 days. In sham operated rats (N* = 5*), only the laminectomy was performed. In addition, injured untreated animals (N* = 2*) were included and sacrificed at day 5 post-injury.

Post-operatively, an antibiotic (Borgal, Hoechst, AG) was administered once/daily for 7 days to prevent urinary tract infection, and an analgesic (Temgesic, Schering-Plough, AB) was administered twice/day for 3 days. Bladders were manually expressed twice daily for the first 7 days post-injury and once/day thereafter until bladder function was restored. Residual urine was collected and the volumes recorded for 21 days post-injury. Weights were recorded before injury, day 3 post-injury, and weekly thereafter. Food and water was provided ad libitum, and animals were housed 4/cage in a room with a 12-hour light/dark cycle.

For all of the following experimental procedures, the experimenters were blinded to the treatment groups during the behavioral testing as well as during the histological analysis. To ensure unbiased evaluation, the following steps were taken: one surgeon performed all surgeries, a surgical assistant randomly assigned animals to treatment groups and assigned ID numbers to the rats, and experimenters involved in behavioral experiments did not treat the animals–so they did not know which animals belonged to which treatment group. For histology, the animals were given different ID numbers and the spinal cords of 3 different animals were placed on the same slide. Once staining/IHC analysis was completed, the code was broken and statistical analysis was performed.

### Locomotor Evaluation

The Basso, Beattie, Bresnahan (BBB) locomotor rating scale [23] was used to evaluate hind limb locomotor function in an open field weekly for 8 weeks. All experimenters involved with BBB evaluation were blinded to treatment group identity.

### Sensorimotor Evaluation

The contact plantar placement test was used to assess sensorimotor integration weekly, beginning 1 week post-injury. Briefly, the dorsal surface of each hind paw was lightly touched against the edge of a standard laboratory bench, and the ability of the rat to make plantar contact with the bench was scored by the number of times this response was elicited during three trials for each hind limb individually (max. score  = 6). Data presented as the sum of left and right responses. All experimenters involved with BBB evaluation were blinded to treatment group identity.

### Immunohistochemistry, Hematoxylin and Eosin, and Luxol Fast Blue Staining

Albumin (Abcam), biotinylated anti-rat IgG antibodies (Santa Cruz Biotechnology), fibronectin (Abcam), and claudin-5 (Zymed) antibodies were used to evaluate vascular permeability and BSCB integrity [47], [48]. Antibodies to CD68 or CD11b (AbD Serotec) were used to evaluate activated and non-activated macrophages/microglia, respectively. GFAP antibodies (Dako and Sigma-Aldrich) were used to evaluate astrocyte reactivity. Ki67 antibodies (Pharmingen and Novocastra) were used to evaluate cell proliferation. PDGFR-α (Cell Signaling), PDGFR-β (Cell Signaling), and NG2 (Chemicon) antibodies were used to visualize astrocytes, pericytes, and progenitor cells, respectively. CS-56 antibodies (Sigma) were used to evaluate CSPG deposition. Neurofilament antibodies (SMI-312, Abcam) were used to evaluate axon sparing in white matter. MPO antibodies (Bio Site) were used to evaluate neutrophil infiltration into the injured spinal cord. Biotinylated *lycopersicon esculentum* (tomato) lectin (Vector Laboratories) and occludin-1 (Zymed) or podocalyxin (R&D Systems) antibodies were used as endothelial cell markers. NeuN antibodies (Chemicon) were used as neuronal markers. DAPI (Invitrogen) was used to identify nuclei. For PDGFR-α and -β immunolabeling, antigen retrieval for 15 minutes at 95°C with high pH retrieval buffer (Dako) was required. Sections were incubated for 1 hour at room temperature with either Alexa fluorophores (Molecular Probes) or Cy3-labeled avidin. Bound antibodies were visualized using epifluorescence microscopy (Nikon Eclipse1000) and photographed (Spot camera, Spot Advance software, Tekno optik), or visualized and photographed using confocal microscopy (Zeiss 710 and LSM software).

For Hematoxylin and Eosin staining, cryosections were stained with Mayer’s hematoxylin. Thereafter, sections were incubated in 0.2% eosin solution (Histolab). For luxol fast blue staining, cryosections were stained with luxol fast blue solution (Sigma Aldrich).

### ELISA

Cytokines (IL-1β, IL-6, IL-8, TNF-α) in the supernatant was analyzed using a 9-plex ultra-sensitive elisa kit from Mesoscale (Mesoscale, Gaithersburg, Maryland). After blocking with BSA for 30 minutes the supernatant was added to the plate and incubated overnight at 4°C. After washing, the plate was incubated with MSD-SULFO-TAG labeled secondary antibody for 2 hours, washed, and analyzed using a Mesoscale plate reader.

### Transgenic Mice

*PDGFRα* ^+/GFP^ mice on C57BL/6J genetic background were perfused with 4% PFA/PBS under deep Hypnorm/Dormicum anesthesia.

### Stereology

Unbiased stereology was used to determine the volumes spared tissue and spinal cysts in a 7 mm segment centered on the injury site and a 7 mm segment immediately caudal to the injury segment stained with Hematoxylin and Eosin. We also used unbiased stereology to quantify the volume of spared myelin within the injury site in sections stained with luxol fast blue. With both stains, a total of 9 sections were analyzed per animal for each of the spinal segments. Stereological analysis was based on the Cavalieri Principle (Stereologer, SPA Inc., MD) using the three dimensional optical dissector [49]. Results are reported as mean volume ± SEM.

### Statistical Evaluation

For comparisons of BBB scores, residual urine volumes, CPP scores, tissue preservation volumes, and weight gain, Repeated measures ANOVA was performed with Bonferroni post-hoc test. The confidence interval was set at 95%. Data are presented as mean ± SEM, with *P*-values <0.05 considered significant.

For immunofluorescence, patterns were photographed; and from each animal, 3 micrographs/spinal cord segment were taken for each animal of each spinal cord regions assessed. The micrographs were analyzed using ImageJ (NIH). The proportions of stained area/total area were compared between treatment groups. The proportion of proliferating cells was evaluated from co-immunolabeled sections by manual counting. Data are presented as mean ± SD and *P*-values were obtained using Student’s *t*-test. *P*-values <0.05 were considered significant.

## Supporting Information

Figure S1 **Scatter plot representation of functional outcome at day 56 post-injury.** Hindlimb locomotor function assessed by the BBB locomotor rating scale. The red line indicates BBB score 9, when weight-support begins. Scores above 9 represent walking, while scores below 9 represent no walking. (**B**) Contact plantar placement (CPP) test of sensorimotor function. (**C**) Bladder residual urine volumes. Imatinib (N = 10) compared to PBS (N = 10). Data presented as the mean ± SD.
(TIF)

Figure S2 **Effects of 30 minute delayed treatment on injury-induced weight loss.** Results from a repeated measures ANOVA revealed that imatinib had significant treatment (P<0.001) and time effects (P<0.001; imatinib, N = 10; PBS, N = 10). Data presented as the mean ± SEM. 0 =  day of spinal cord injury.
(TIF)

Figure S3 **Myelin preservation.** Representative micrographs of luxol fast blue staining within in the injury site at day 56 post-injury in the spinal cords of (**A**) PBS treated rats. (**B**) imatinib treated rats. (**C**) Quantification of the volume of luxol fast blue stained tissue within the injury site at day 56 post-injury (p = 0.0504; imatinib, N = 6; PBS, N = 6). Data presented as the mean ± SD.
(TIF)

Figure S4 **Proliferation.** Representative micrographs of co-labeling of (**A**) CD68, (**B**) CD11b, (**C**) PDGFR-α, (**D**) PDGFR-β, and (**E**) NG2 with Ki67 at day 5 post-injury in rats treated with PBS starting 30 minutes after injury. (**F**) Quantification of proliferating cells assessed through Ki67-positive area within the injury site and in spinal segments 7 and 14 mm rostrally and caudally from the injury 5 days post-injury in rats treated with either 30 minute delayed PBS or imatinib treatment (imatinib, N = 4; PBS, N = 4). (**G**) Scatter plot of Ki67-positive area within the injury site at day 5 post-injury (imatinib, N = 4; PBS, N = 4). Arrows indicate co-labeled cells. Data presented as mean ± SD: *P<0.05 and ***P<0.001. Scale bars: (**A, B**) 10 µm, (**C-E**) 20 µm.
(TIF)

Figure S5 **Macrophage/microglia co-expression with PDGFR-α.** Representative micrographs of CD68 expression with (**A**) PDGFR-α and (**B**) NG2 expression within the injury site at day 7 post-injury. Representative micrographs of CD11b co-localization with NG2 at (**C**) 14 mm caudal and (**D**) 7 mm caudal to the injury site and (**E**) within the injury site. Scale bars: 100 µm.
(TIF)

Figure S6 **Scatter plot respresentation of CS56 and NG2 expression.** (**A**) Quantification of CS56 immunoreactivity and (**B**) NG2 immunoreactivity within the injury site at day 5 post-injury (imatinib, N = 4; PBS, N = 4). Data presented as mean ± SD.
(TIF)

Figure S7 **PDGFR expression patterns.** PDGFR expression at 24 hours post-injury in rats pre-treated with imatinib or PBS 4 hours before injury. (**A**) Representative micrographs of PDGFR-α expression in sham operated, PBS, and imatinib treated rats. (**B**) Quantification of the area of PDGFR-α immunoreactivity. (**C**) Representative micrographs of PDGFR-β expression in sham operated, PBS, and imatinib treated rats. (**D**) Quantification of the area of PDGFR-β immunoreactivity. Imatinib (n = 4), PBS (n = 4), Sham (n = 1). Data presented as mean ± SD: *P<0.05, **P<0.01, and ***P<0.001. Scale bars: 100 µm.
(TIF)

Figure S8 **PDGFR co-expression.** PDGFR expression in uninjured and injured mouse and rat spinal cords at day 5 post-injury. (**A**) Representative micrographs of PDGFR-α promotor driven GFP and NeuN/DAPI immunoreactivity in non-injured mouse spinal cord. (**B**) Representative micrographs of PDGFR-α/NeuN/DAPI immunoreactivity in noninjured mouse spinal cord. (**C**) Representative micrographs of PDGFR-β/NeuN/DAPI immunoreactivity in noninjured mouse spinal cord. (**D**) Representative micrographs of PDGFR-α/NeuN/DAPI immunoreactivity in injured rat spinal cord. (**E**) Representative micrographs of PDGFR-β/NeuN/DAPI immunoreactivity in injured rat spinal cord. Scale bars: 10 µm (**A-C**), 50 µm (**D,E**).
(TIF)
