# Supplementary figures and images for: Imatinib Enhances Functional Outcome after Spinal Cord Injury
Source: PLoS One. 2012 Jun 19;7(6):e38760. doi: 10.1371/journal.pone.0038760 (PMC3378614; doi:10.1371/journal.pone.0038760)

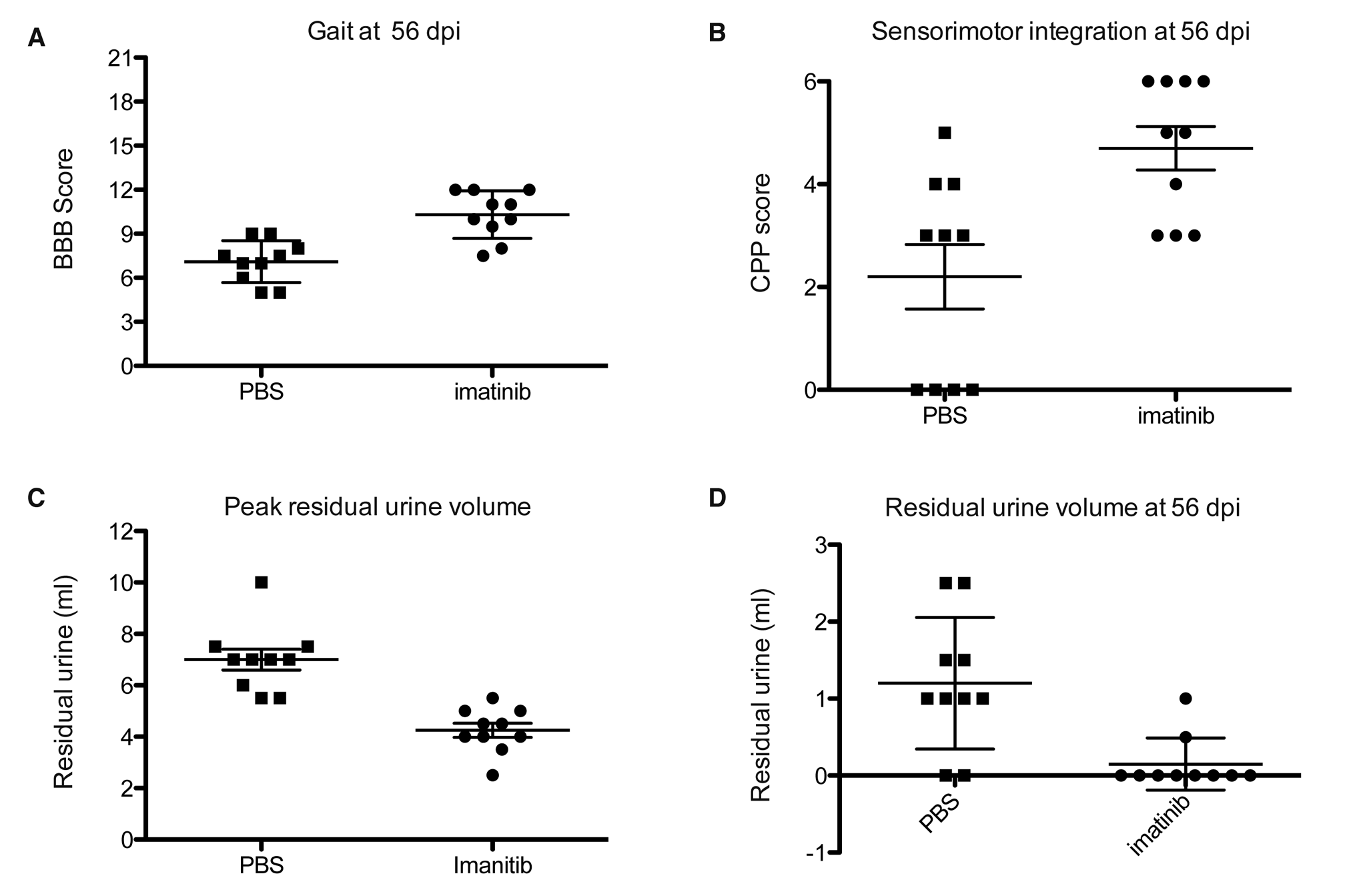

Supplement: Figure S1 — Scatter plot representation of functional outcome at day 56 post-injury. Hindlimb locomotor function assessed by the BBB locomotor rating scale. The red line indicates BBB score 9, when weight-support begins. Scores above 9 represent walking, while scores below 9 represent no walking. (B) Contact plantar placement (CPP) test of sensorimotor function. (C) Bladder residual urine volumes. Imatinib (N = 10) compared to PBS (N = 10). Data presented as the mean ± SD. (TIF) [file pone.0038760.s001.tif]

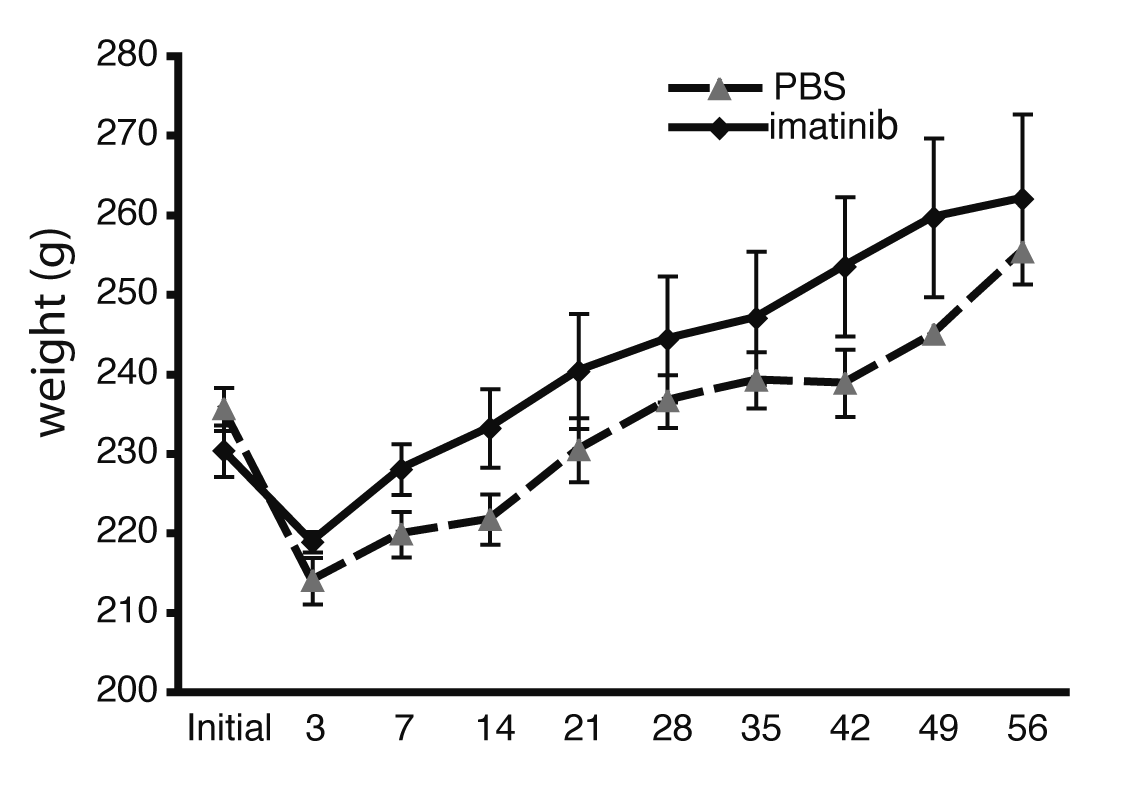

Supplement: Figure S2 — Effects of 30 minute delayed treatment on injury-induced weight loss. Results from a repeated measures ANOVA revealed that imatinib had significant treatment (P<0.001) and time effects (P<0.001; imatinib, N = 10; PBS, N = 10). Data presented as the mean ± SEM. 0 = day of spinal cord injury. (TIF) [file pone.0038760.s002.tif]

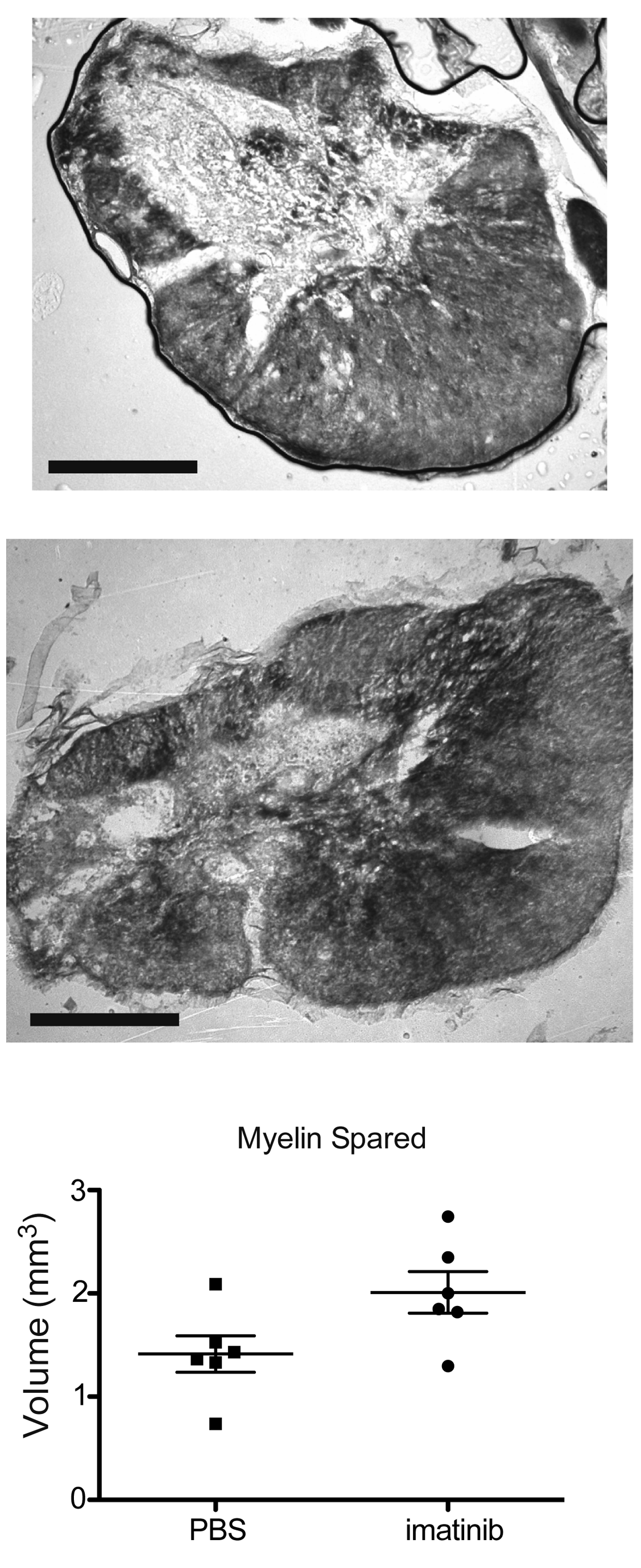

Supplement: Figure S3 — Myelin preservation. Representative micrographs of luxol fast blue staining within in the injury site at day 56 post-injury in the spinal cords of (A) PBS treated rats. (B) imatinib treated rats. (C) Quantification of the volume of luxol fast blue stained tissue within the injury site at day 56 post-injury (p = 0.0504; imatinib, N = 6; PBS, N = 6). Data presented as the mean ± SD. (TIF) [file pone.0038760.s003.tif]

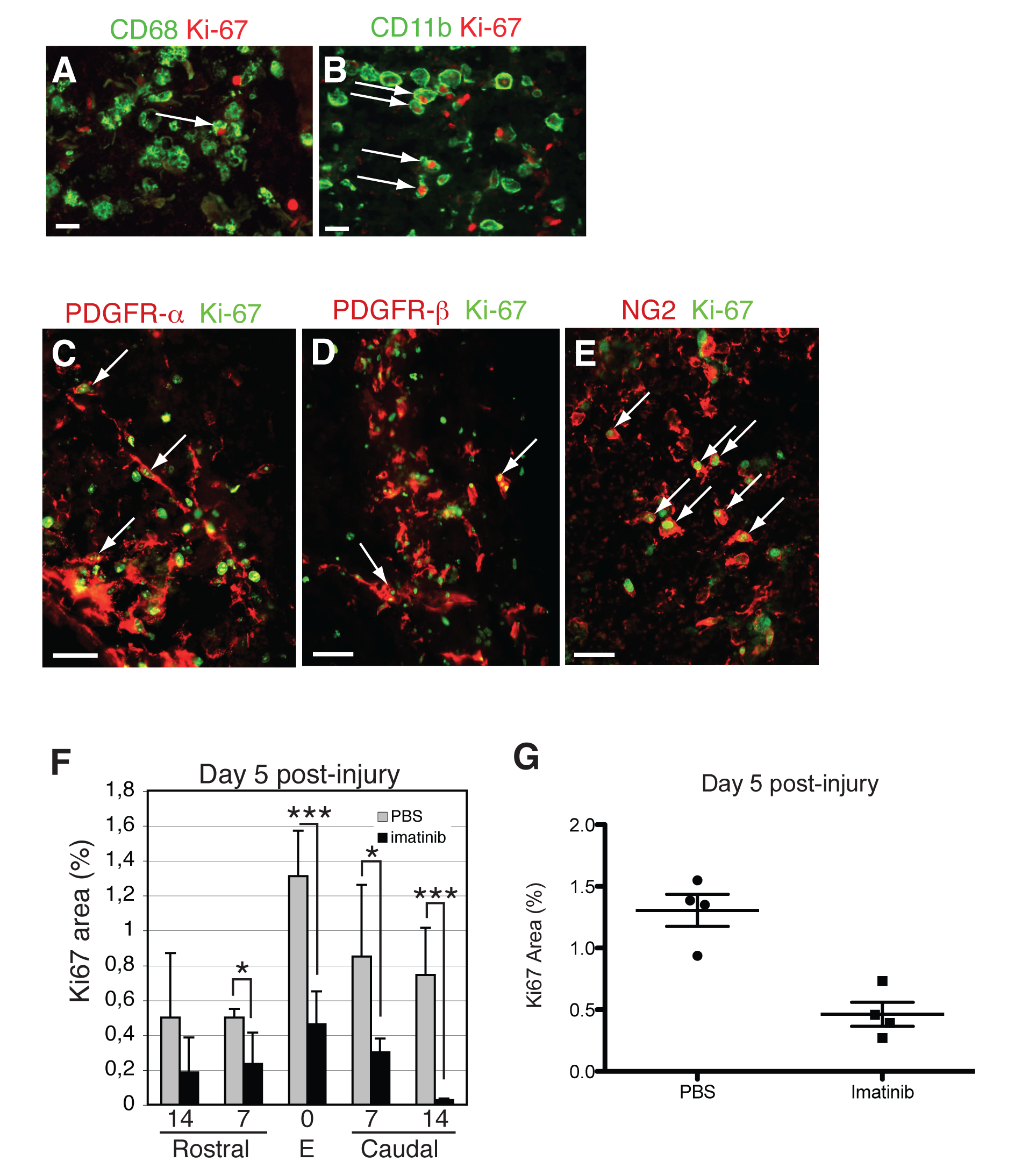

Supplement: Figure S4 — Proliferation. Representative micrographs of co-labeling of (A) CD68, (B) CD11b, (C) PDGFR-α, (D) PDGFR-β, and (E) NG2 with Ki67 at day 5 post-injury in rats treated with PBS starting 30 minutes after injury. (F) Quantification of proliferating cells assessed through Ki67-positive area within the injury site and in spinal segments 7 and 14 mm rostrally and caudally from the injury 5 days post-injury in rats treated with either 30 minute delayed PBS or imatinib treatment (imatinib, N = 4; PBS, N = 4). (G) Scatter plot of Ki67-positive area within the injury site at day 5 post-injury (imatinib, N = 4; PBS, N = 4). Arrows indicate co-labeled cells. Data presented as mean ± SD: *P<0.05 and ***P<0.001. Scale bars: (A, B) 10 µm, (C-E) 20 µm. (TIF) [file pone.0038760.s004.tif]

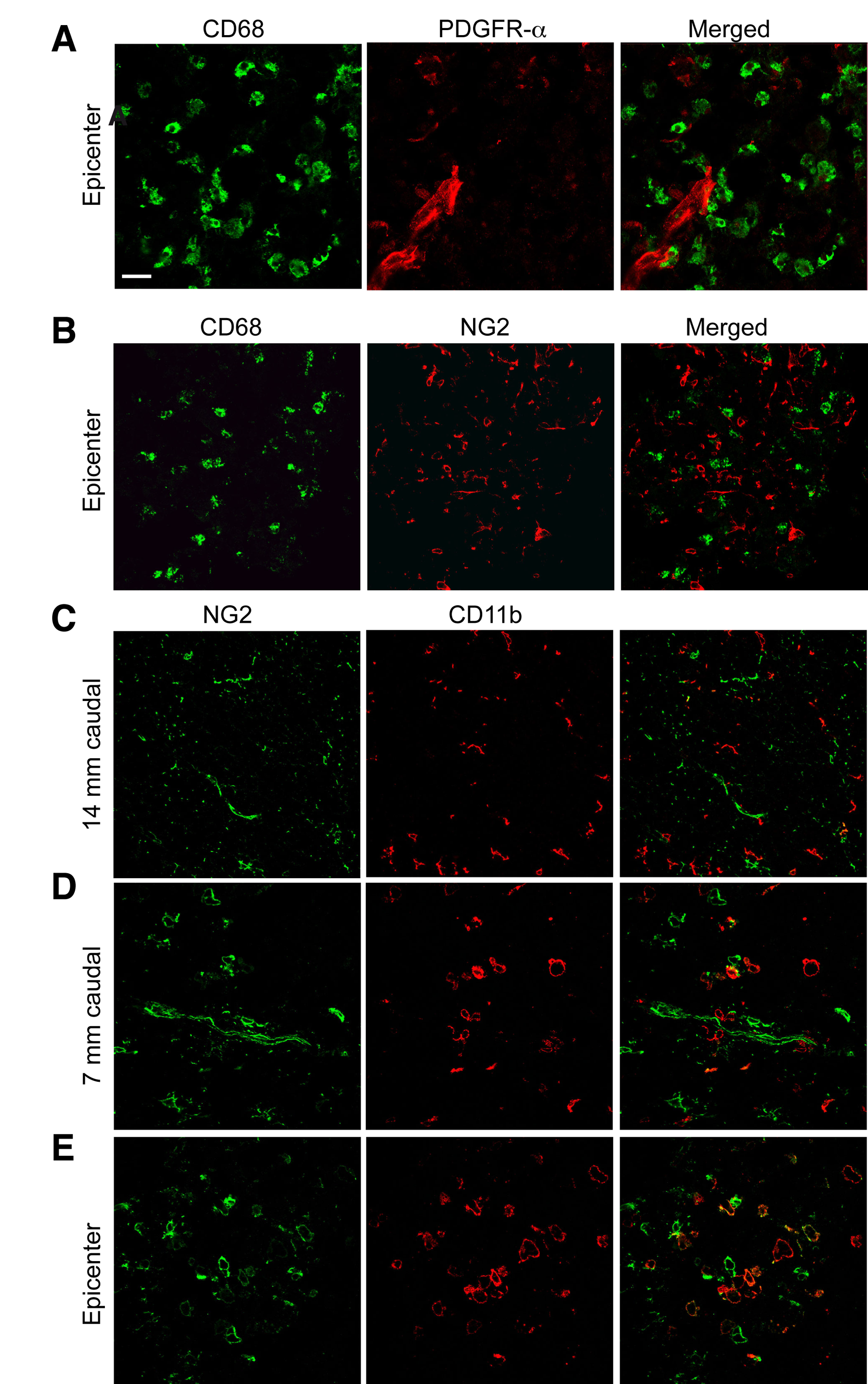

Supplement: Figure S5 — Macrophage/microglia co-expression with PDGFR-α. Representative micrographs of CD68 expression with (A) PDGFR-α and (B) NG2 expression within the injury site at day 7 post-injury. Representative micrographs of CD11b co-localization with NG2 at (C) 14 mm caudal and (D) 7 mm caudal to the injury site and (E) within the injury site. Scale bars: 100 µm. (TIF) [file pone.0038760.s005.tif]

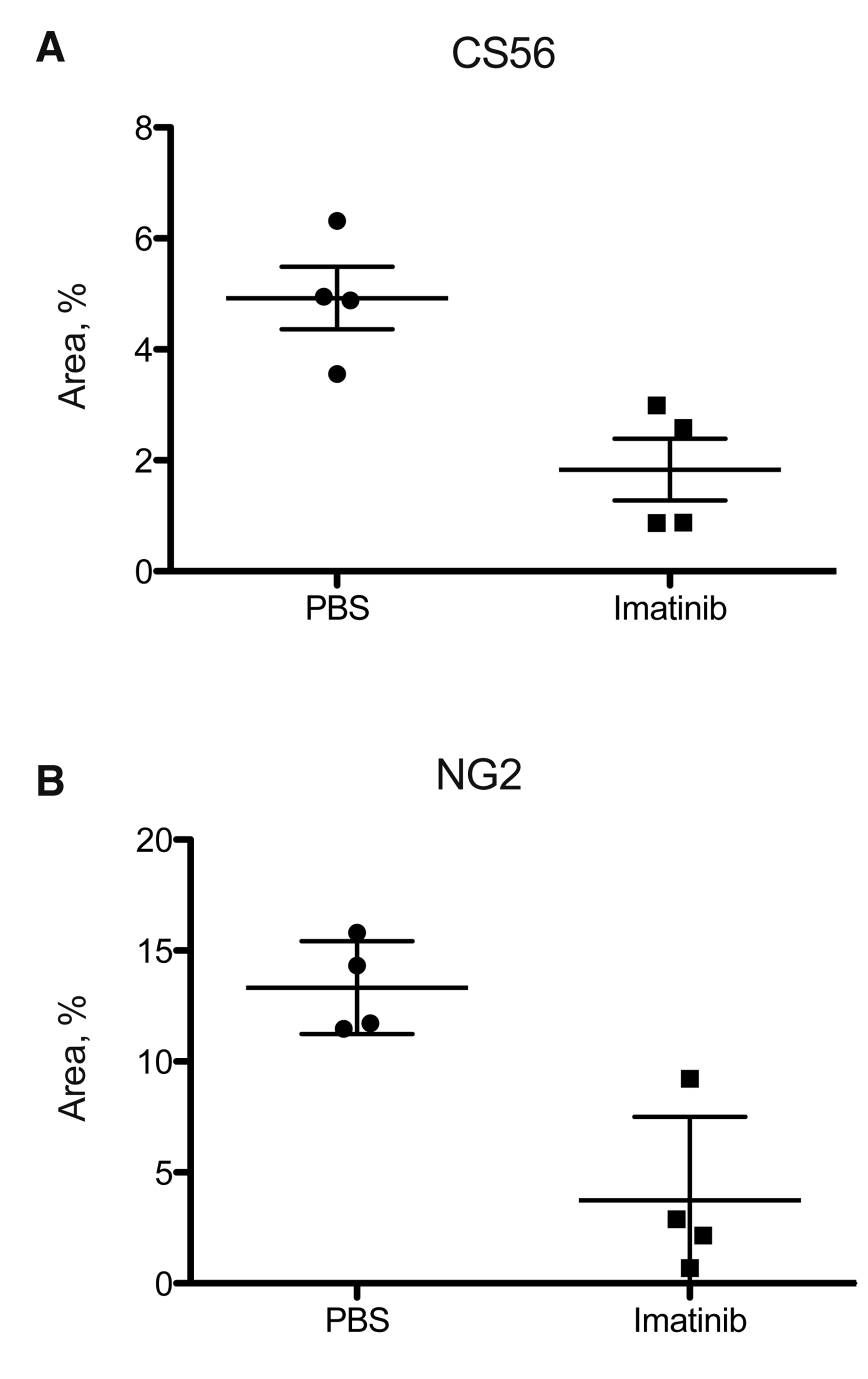

Supplement: Figure S6 — Scatter plot respresentation of CS56 and NG2 expression. (A) Quantification of CS56 immunoreactivity and (B) NG2 immunoreactivity within the injury site at day 5 post-injury (imatinib, N = 4; PBS, N = 4). Data presented as mean ± SD. (TIF) [file pone.0038760.s006.tif]

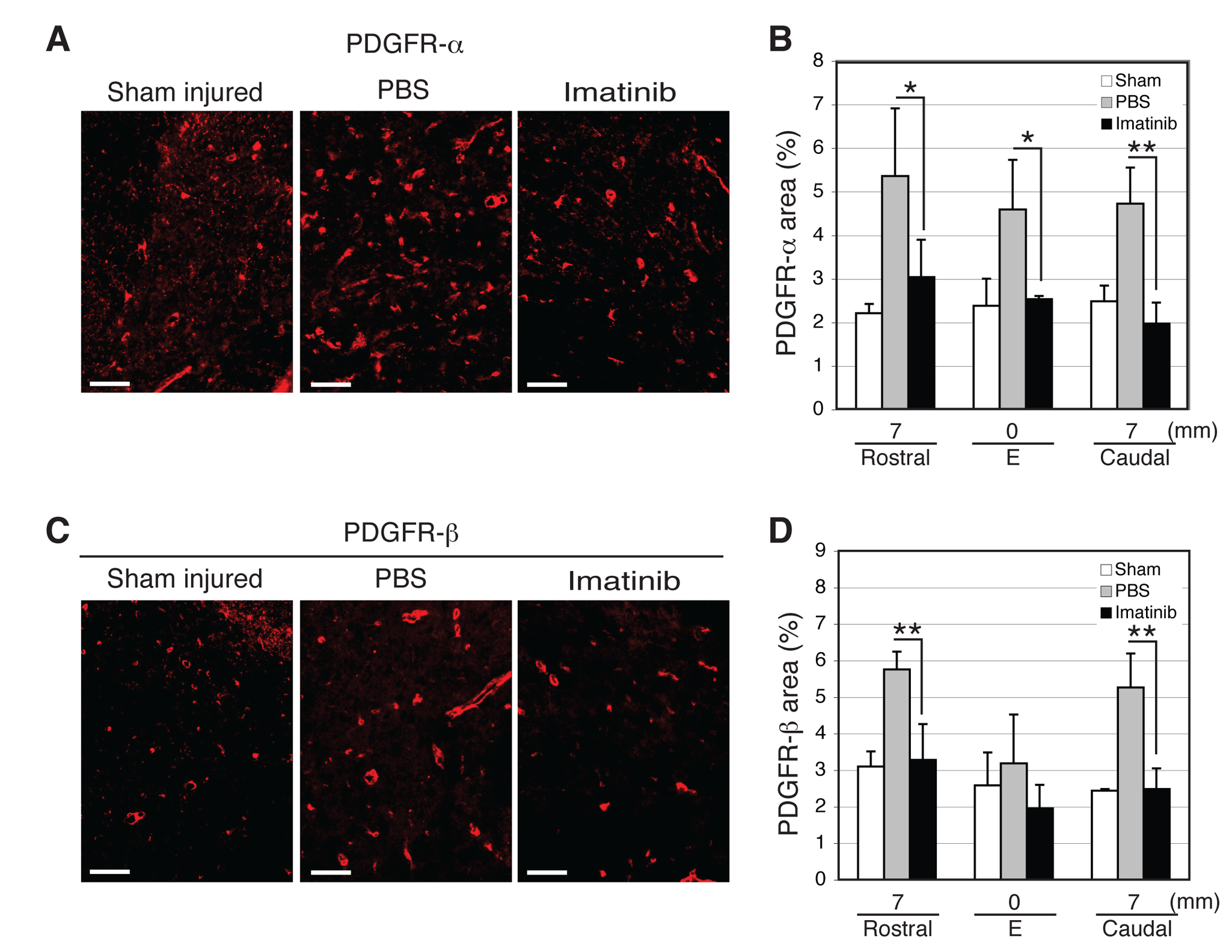

Supplement: Figure S7 — PDGFR expression patterns. PDGFR expression at 24 hours post-injury in rats pre-treated with imatinib or PBS 4 hours before injury. (A) Representative micrographs of PDGFR-α expression in sham operated, PBS, and imatinib treated rats. (B) Quantification of the area of PDGFR-α immunoreactivity. (C) Representative micrographs of PDGFR-β expression in sham operated, PBS, and imatinib treated rats. (D) Quantification of the area of PDGFR-β immunoreactivity. Imatinib (n = 4), PBS (n = 4), Sham (n = 1). Data presented as mean ± SD: *P<0.05, **P<0.01, and ***P<0.001. Scale bars: 100 µm. (TIF) [file pone.0038760.s007.tif]

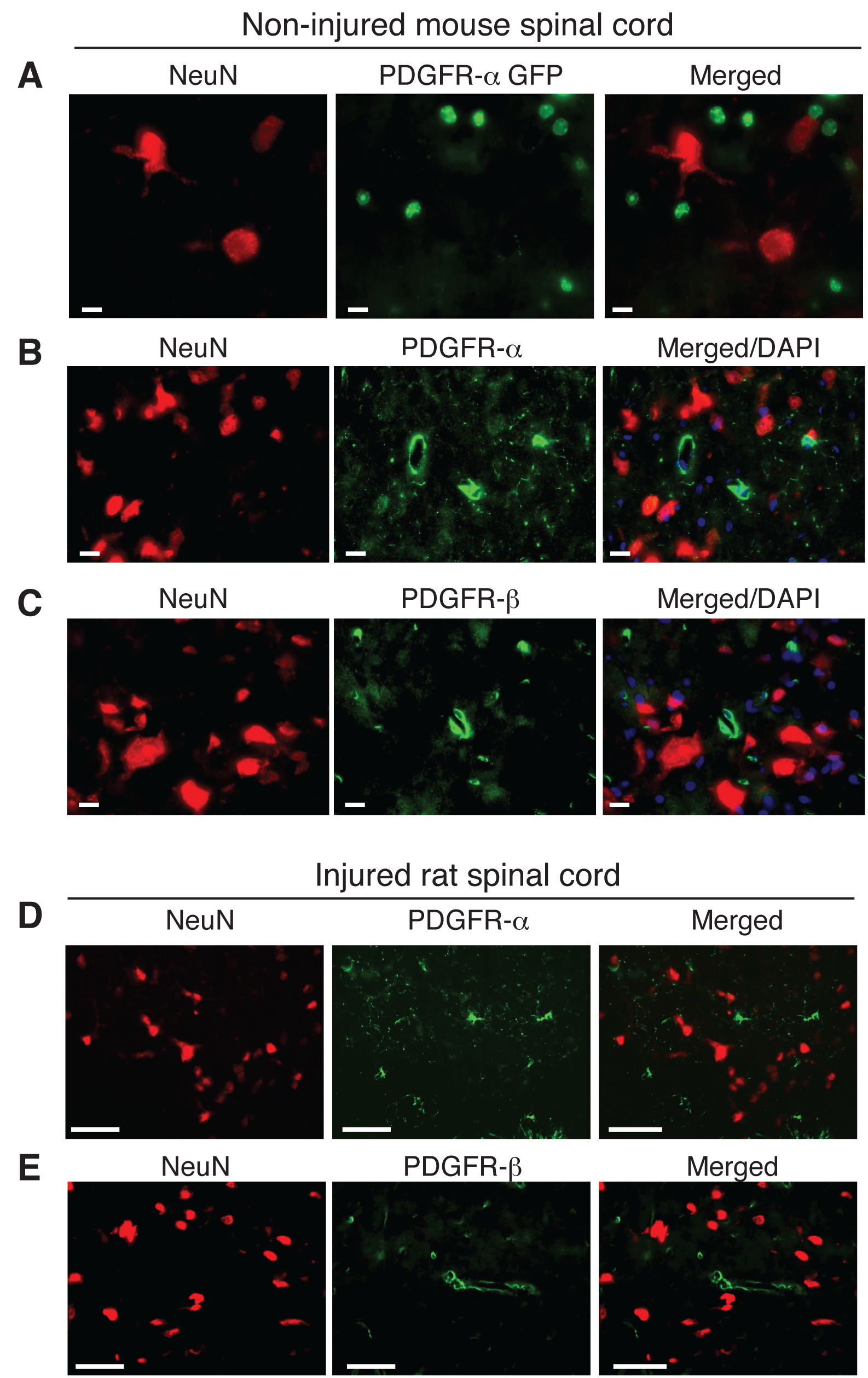

Supplement: Figure S8 — PDGFR co-expression. PDGFR expression in uninjured and injured mouse and rat spinal cords at day 5 post-injury. (A) Representative micrographs of PDGFR-α promotor driven GFP and NeuN/DAPI immunoreactivity in non-injured mouse spinal cord. (B) Representative micrographs of PDGFR-α/NeuN/DAPI immunoreactivity in noninjured mouse spinal cord. (C) Representative micrographs of PDGFR-β/NeuN/DAPI immunoreactivity in noninjured mouse spinal cord. (D) Representative micrographs of PDGFR-α/NeuN/DAPI immunoreactivity in injured rat spinal cord. (E) Representative micrographs of PDGFR-β/NeuN/DAPI immunoreactivity in injured rat spinal cord. Scale bars: 10 µm (A-C), 50 µm (D,E). (TIF) [file pone.0038760.s008.tif]
